# Supplementary material for: Protein disulfide isomerase cleaves allosteric disulfides in histidine-rich glycoprotein to regulate thrombosis
Source: Nat Commun. 2024 Apr 11;15:3129. doi: 10.1038/s41467-024-47493-0 (PMC11009332; doi:10.1038/s41467-024-47493-0)
Supplement: Supplementary file 1 — Supplementary Information [file 41467_2024_47493_MOESM1_ESM.pdf]

Supplemental materials for Lv *et al.*

Supplemental Figures

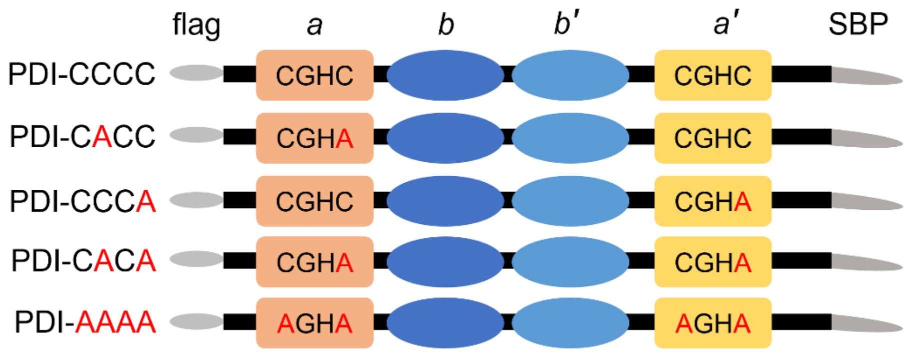

Fig. S1: The schematic depicting the PDI variants used in the mechanism-based kinetic trapping. The structure of PDI includes 2 catalytically active domains *a* and *a′* with each containing a Cys-Gly-His-Cys motif (denoted as CCCC). Mutation of the C-terminal Cys to Ala in the Cys-Gly-His-Cys motif in *a* domain (denoted as CACC), in *a′* domain (denoted as CCCA), or both domains (denoted as CACA), results in the trapping mutants of PDI which form stabilized transient disulfide-linked complexes with their substrates. Mutation of all the catalytic Cys to Ala (denoted as AAAA) leads to the inert variant of PDI. These variants were cloned into a pT7-FLAG-SBP vector to add an N-terminal Flag epitope for detection by immunoblotting and a C-terminal Streptavidin Binding Peptide (SBP) for purification by chromatography.

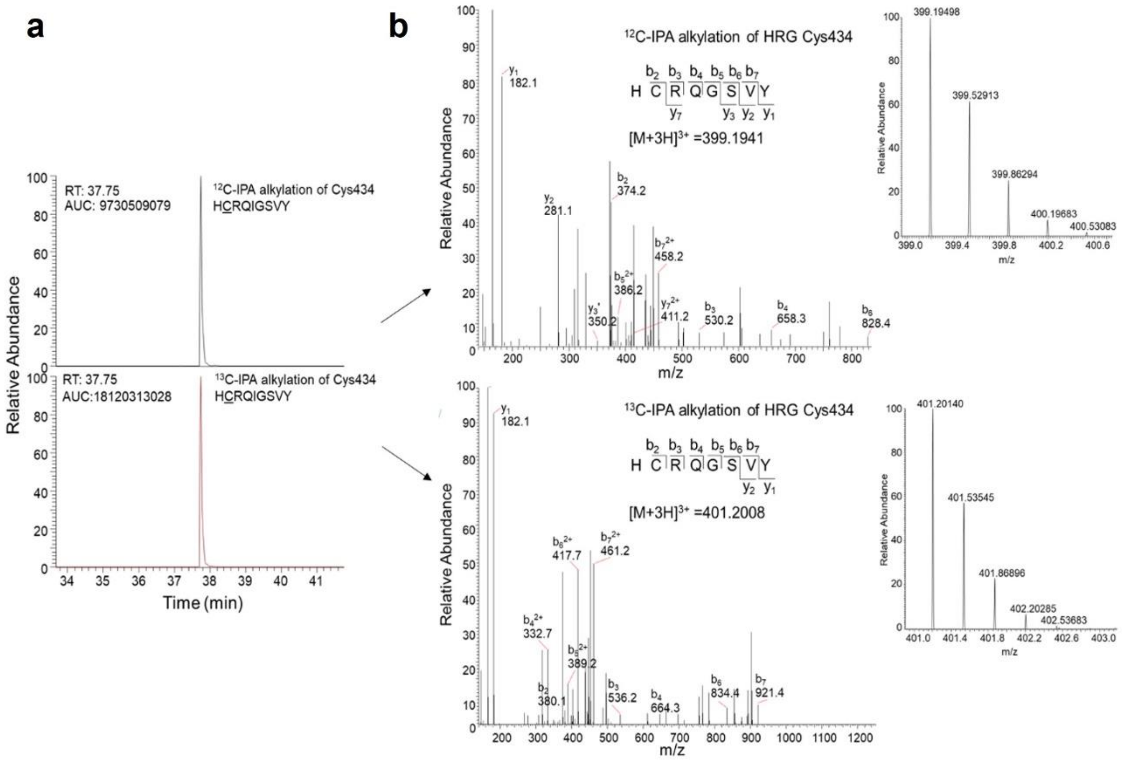

Fig. S2: Redox quantification of differential cysteine alkylation mass spectrometry output. (**a**) HPLC resolution of C434 containing peptide from purified HRG from human plasma. The peaks belonging to peptides ^{12}C-IPA or ^{13}C-IPA labelled peptides containing cysteines of interest were identified and the AUC recorded. (**b**) The identity of a peak was verified in tandem mass spectrometry. Percentage of reduction is calculated as the AUC of the ^{12}C-IPA labelled peptide divided by the sum of the AUC of ^{12}C-IPA and ^{13}C-IPA.

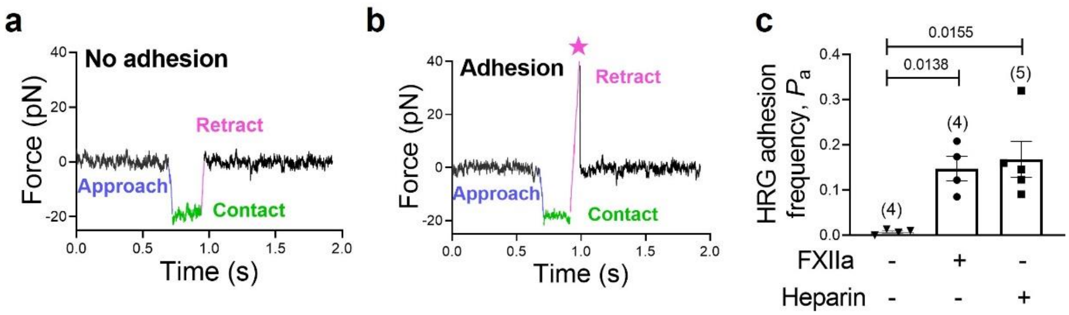

Fig. S3: Biomembrane Force Probe (BFP) detection of the interactions of HRG with heparin and FXIIa. Representative BFP traces of events with no adhesion (**a**) or with adhesion occurrence (**b**). The force trace marked with a star represents the occurrence of adhesion between the Probe-Target pair. (**c**) The interactions of HRG-immobilized beads with different Target beads as indicated. The adhesion frequencies (P_a) were measured with contact time (t_c) at 0.2 s. Only minimal adhesion frequency (< 2%) was detected if the Target bead was not coated, demonstrating the specificity of the interactions of HRG with heparin and FXIIa during the BFP assay. The number of probe-target pairs (n value) analyzed in each group was indicated above the bars. The data are presented as mean values ± SEM and analyzed by Welch's ANOVA test (c). Source data are provided as a Source Data file.

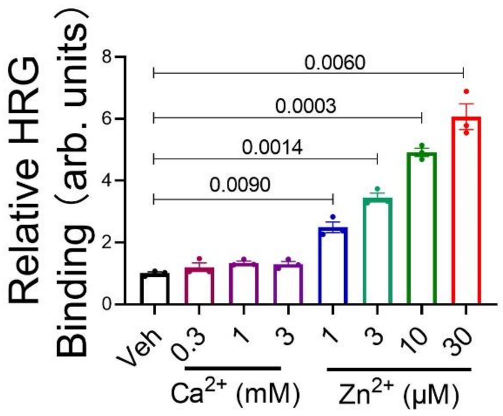

Fig. S4: The effect of Ca^{2+} and Zn^{2+} on the binding of HRG on heparin. Purified human HRG was incubated on microtiter plates pre-coated with heparin in the presence of indicated concentrations of Ca^{2+} (0.3, 1, 3 mM) or Zn^{2+} (1, 3, 10, 30 μM). The bound HRG was determined by ELISA using HRP-conjugated mouse-anti human HRG (n = 3 independent samples). The data were normalized to the group without cation. Veh, vehicle. The data are presented as mean values ± SEM and analyzed by Welch's ANOVA test. Source data are provided as a Source Data file.

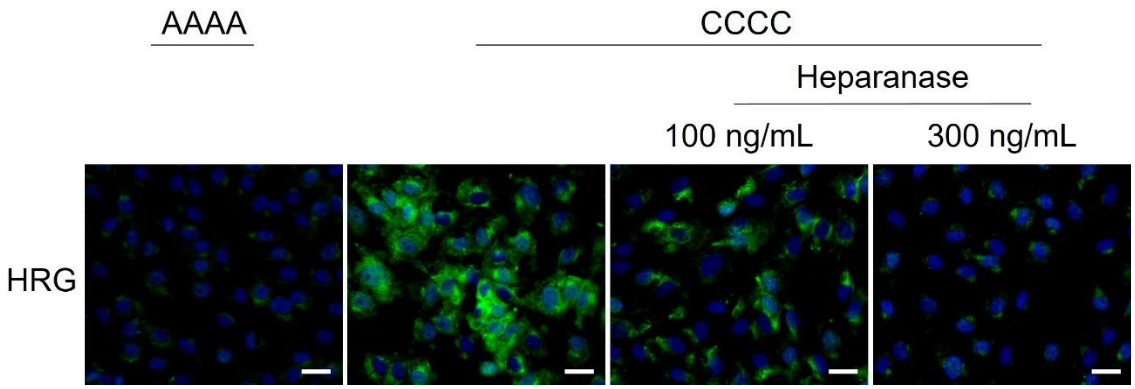

Fig. S5: The effect of heparanase on HRG binding on endothelial cells. Human plasma pre-treated with recombinant human PDI-CCCC or PDI-AAAA was incubated in the presence of Zn^{2+} (10 μM) on HUVECs pre-treated with or without different concentrations of heparanase (100 and 300 ng/mL). The binding of HRG on the cell surface were determined by immunofluorescence using Alexa-488-conjugated antibodies (scale bar: 20 μm).

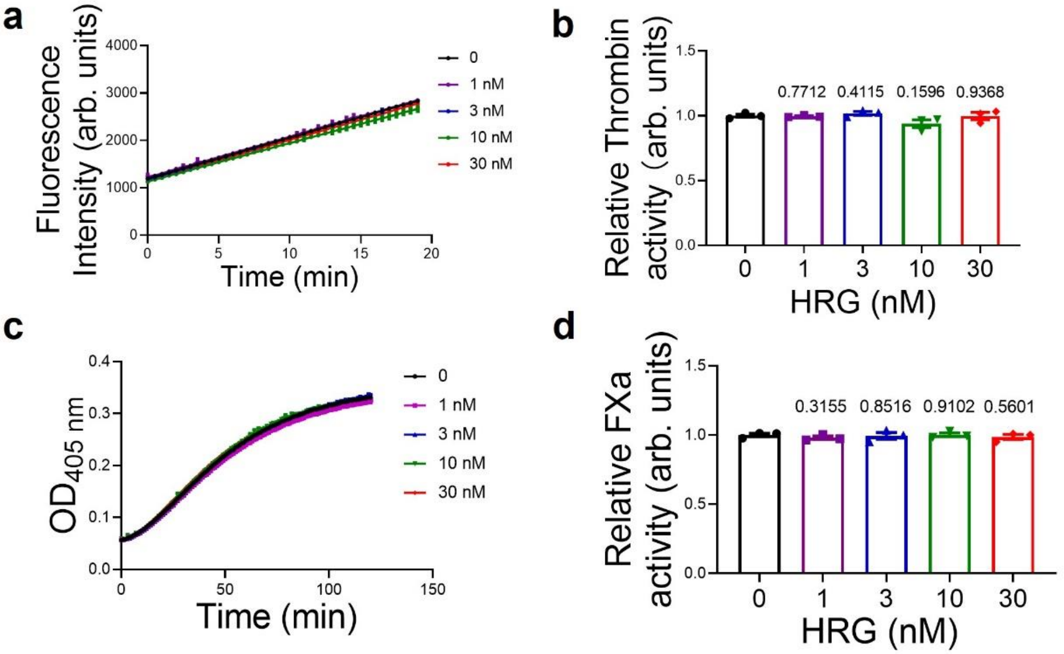

Fig. S6: The effect of HRG on the activities of thrombin and FXa. (**a-b**) Human α-thrombin was mixed with indicated concentrations of purified human HRG. The activity of thrombin was determined as the cleavage of Z-Gly-Gly-Arg-AMC·HCl. The fluorescence (**a**) was monitored on a plate reader at 390 nm/460 nm (Ex/Em) and the reaction slope (**b**) was calculated for each time point (n = 3 independent samples). (**c-d**) Human FX was mixed with FXa substrate Biophen CS-11, TF and indicated concentrations of purified human HRG in the presence of 5 mM $CaCl_2$. The reaction was initiated by the addition of human FVIIa. The OD at 405 nm (**c**) was monitored on a plate reader and the reaction slope (**d**) was calculated for each time point (n = 3 independent samples). The data are presented as mean values ± SEM and analyzed by Welch's ANOVA test (b and d). Source data are provided as a Source Data file.

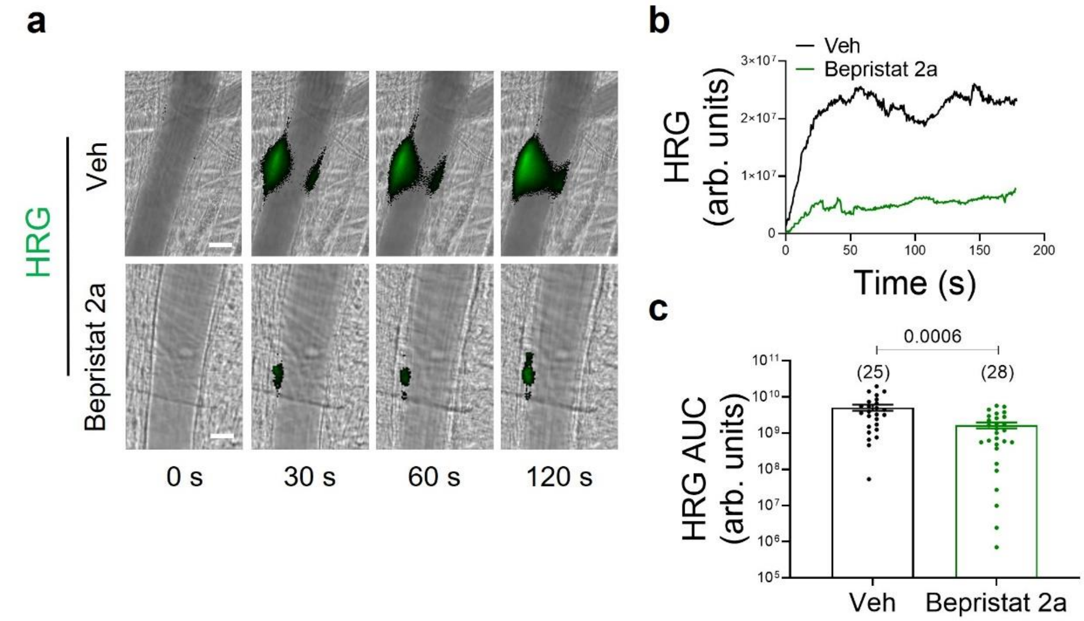

Fig. S7: The effect of a PDI inhibitor Bepristat 2a on HRG accumulation during thrombus formation. (**a**) Representative images of HRG incorporation (Green), visualized using Alexa-488-conjugated anti-HRG, at indicated time points following laser injury in the cremaster arterioles in mice treated with vehicle or Bepristat 2a (15 µg/g body weight) (scale bar: 30 µm). The median fluorescence intensity (**b**) and the area under the curve (AUC) (**c**) of HRG were analyzed from each individual thrombus in the two groups. The number of thrombi (n value) analyzed in each group was indicated above the bars. Veh, vehicle. The data are presented as mean values ± SEM and analyzed by two-tailed Mann-Whitney *U*-test. Source data are provided as a Source Data file.

Supplemental Tables

Table S1: HRG peptides analyzed to determine disulfide bond redox state. Peptides were detected by both Byonic and Mascot analysis software, and confirmed by MS/MS with errors < 6 ppm. Only peptides with peak areas > 10 million for a given Cys were included in the analysis.

Disulfide bond	Cys	Peptide sequence*	Peptide score	Peptide error (ppm)
C6-C486	6	VSPTD**C**SAVEPEAEKAL	406	3.8
		VSPTD**C**SAVEPEAEKALDL	449.8†	0.7
	486	HKHPLKPDNQPFPQSVSES**C**PGKF	530.8†	2.3
		KHPLKPDNQPFPQSVSES**C**PGKF	380.9†	5.0
		KPDNQPFPQSVSES**C**PGKF	61	3.29
		PQSVSES**C**PGKF	638.5†	4.6
C60-C71	60	VLDVQESD**C**SVL	48	2.71
		LVLDVQESD**C**SVL	45	5.04
	71	WND**C**EPPDSRRPSEIVIGQ**C**KVIATRH	563.1†	3.3
		ND**C**EPPDSRRPSEIVIGQ**C**KVIATRH	368.8†	4.0
C87-C108	87	WND**C**EPPDSRRPSEIVIGQ**C**KVIATRH	563.1†	3.3
		ND**C**EPPDSRRPSEIVIGQ**C**KVIATRH	368.8†	4.0
	108	RVIDF<u>N</u>(deamidated)**C**TTSSVSSAL	82	1.41
		<u>N</u>(deamidated)**C**TTSSVSSAL	234.8†	3.5
C185-C399	185	SVRN**C**PRHH	410.2†	3.3
		SVRN(deamidated)**C**PRHHFPRHPNVF	175.1†	2.6
	399	DFQDYGP**C**DPPPHNQGH	594.8†	-0.7
		GP**C**DPPPH	315.1†	0.3
		GP**C**DPPPHNQGH	450.7†	-0.2
C200-223	200	GF**C**RADL	324.8†	4.6
		GF**C**RADLF	348.9†	1.6
		GF**C**RADLFY	357.8†	1.5
		**C**RADL	237†	1.9
		**C**RADLF	250.2†	1.7
		**C**RADLFY	299.1†	3.5
		**C**RADLFYDVEAL	53	1.97
	223	VIN**C**EVFDPQEH	518.7†	0.9
		VIN**C**EVFDPQEHENINGVPPH	530.7†	2.4
		VIN**C**EVFDPQEHENINGVPPHLGHPF	799.1†	0.9
		VIN**C**EVFDPQEHENIN(deamidated)GV--PPHLGHPF	628.2†	0.9
		VIN**C**EVF	132.1†	1.8
C306-C309	306, 309	S**C**SS**C**QHATF	580.3†	2.5
		S**C**SS**C**QH	288.3†	4.63
C390-C434	390	CHDFQY	287.2†	2.0
	434	H**C**RQIGSVY	38	1.71
		**C**RQIGSVY	379.8†	4.7
C409-C410	409, 410	**CC**HGH	160.1†	3.4
		**CC**HGHGPPPGH	418.9†	2.9
		**CC**HGHGPPPGHL	536.3†	1.2

*Cys was labelled with ^{12}C-iodoacetanilide or ^{13}C-iodoacetanilide and is in bold. Deamidation as a result of deglycosylation by PNGase F is underlined.

†Score and error were determined using Byonic software using HRG as reference.

Table S2: HRG disulfide pairs mapped by mass spectrometry using disulfide-linked peptides. Peptides were detected by Byonic analysis software for disulfide-linked peptides.

Disulfide bond	Disulfide-linked peptide sequence*	Xlink score	Peptide error (ppm)	*P*-value#
C6-C486	VSPTD**C**SAVEPEAEKAL<br>HKHPLKPDNQPFPQSVSES**C**PGKF	308.2	2.61	0.00013
C185-C399	SVRN**C**PRH<br>GP**C**DPPPHNQGH	153.8	2.7	7.0×10^{-5}
C200-223	GF**C**RADLF<br>VIN**C**EVFDPQEH	100.9	0.48	6.5×10^{-6}
C306-C309	S**C**SS**C**QHATF	n/a	3.13	1.3×10^{-5}
C390-C434	**C**H<br>**C**RQIGSVY	38	4.1	0.00014
C409-C410	**CC**HGHGPPPGHL	n/a	2.79	1.3×10^{-5}

*Disulfide cysteines are in bold. Intrapeptide disulfide bond has no Xlink score. C60-C71 and C87-C108 were cross-linked to three peptides that cannot be resolved using MS.

#The false discovery rate (FDR) was set at 0.01 and the *P*-value was computed using a method called two-dimensional FDR and a probability distribution with an exponential right-hand tail.
