# Supplementary material for: Protein disulfide isomerase cleaves allosteric disulfides in histidine-rich glycoprotein to regulate thrombosis
Source: Nat Commun. 2024 Apr 11;15:3129. doi: 10.1038/s41467-024-47493-0 (PMC11009332; doi:10.1038/s41467-024-47493-0)
Supplement: Supplementary file 3 — Description of Additional Supplementary Files [file 41467_2024_47493_MOESM3_ESM.pdf]

Description of Additional Supplementary Files

Supplementary Movie 1: The accumulation of HRG and platelets during laser injury-induced thrombus formation in mice treated with or without eptifibatide. HRG and platelets were visualized by Alexa-488-conjugated anti-HRG and Dylight-649-conjugated anti-CD42c, respectively, following laser-induced vessel injury in the cremaster arterioles. Scale bar: 30 µm. The video was exported at a speed of 16 × of the original speed it was recorded.

Supplementary Movie 2: The accumulation of HRG during laser injury-induced thrombus formation in mice treated with Rutin or vehicle. HRG was visualized by Alexa-488-conjugated anti-HRG following laser-induced vessel injury in the cremaster arterioles. Scale bar: 30 µm. The video was exported at a speed of 16 × of the original speed it was recorded.

Supplementary Movie 3: The accumulation of antithrombin during laser injury-induced thrombus formation in mice treated with Rutin or vehicle. Antithrombin was visualized by Alexa-488-conjugated anti-antithrombin following laser-induced vessel injury in the cremaster arterioles. Scale bar: 30 µm. The video was exported at a speed of 16 × of the original speed it was recorded.

Supplementary Movie 4: Platelet accumulation and fibrin generation during laser injury-induced thrombus formation in *F12*$^{-/-}$ mice treated with anti-HRG or control siRNA. Platelets and fibrin were visualized by Dylight-649-conjugated anti-CD42c antibody and Alexa-488-conjugated 59D8 antibody, respectively, following laser-induced vessel injury in the cremaster arterioles. Scale bar: 30 µm. The video was exported at a speed of 16 × of the original speed it was recorded.

Supplementary Movie 5: Platelet accumulation and fibrin generation during laser injury-induced thrombus formation in *WT*, *Hrg*$^{-/-}$, *F12*$^{-/-}$ and *DKO* mice. Platelets and fibrin were visualized by Dylight-649-conjugated anti-CD42c antibody and Alexa-488-conjugated 59D8 antibody, respectively, following laser-induced vessel injury in the cremaster arterioles. Scale bar: 30 µm. The video was exported at a speed of 16 × of the original speed it was recorded.
