# Supplementary material for: Protein disulfide isomerase cleaves allosteric disulfides in histidine-rich glycoprotein to regulate thrombosis
Source: Nat Commun. 2024 Apr 11;15:3129. doi: 10.1038/s41467-024-47493-0 (PMC11009332; doi:10.1038/s41467-024-47493-0)
Supplement: Supplementary file 9 — Reporting Summary [file 41467_2024_47493_MOESM9_ESM.pdf]

nature portfolio

Corresponding author(s): Chao Fang, Ph.D.

Last updated by author(s): Mar 19, 2024

Reporting Summary

Nature Portfolio wishes to improve the reproducibility of the work that we publish. This form provides structure for consistency and transparency in reporting. For further information on Nature Portfolio policies, see our Editorial Policies and the Editorial Policy Checklist.

Statistics

For all statistical analyses, confirm that the following items are present in the figure legend, table legend, main text, or Methods section.

n/a	Confirmed	
☐	☒	The exact sample size (*n*) for each experimental group/condition, given as a discrete number and unit of measurement
☐	☒	A statement on whether measurements were taken from distinct samples or whether the same sample was measured repeatedly
☐	☒	The statistical test(s) used AND whether they are one- or two-sided<br>*Only common tests should be described solely by name; describe more complex techniques in the Methods section.*
☒	☐	A description of all covariates tested
☐	☒	A description of any assumptions or corrections, such as tests of normality and adjustment for multiple comparisons
☐	☒	A full description of the statistical parameters including central tendency (e.g. means) or other basic estimates (e.g. regression coefficient) AND variation (e.g. standard deviation) or associated estimates of uncertainty (e.g. confidence intervals)
☐	☒	For null hypothesis testing, the test statistic (e.g. *F*, *t*, *r*) with confidence intervals, effect sizes, degrees of freedom and *P* value noted<br>*Give P values as exact values whenever suitable.*
☒	☐	For Bayesian analysis, information on the choice of priors and Markov chain Monte Carlo settings
☒	☐	For hierarchical and complex designs, identification of the appropriate level for tests and full reporting of outcomes
☐	☒	Estimates of effect sizes (e.g. Cohen's *d*, Pearson's *r*), indicating how they were calculated

Our web collection on statistics for biologists contains articles on many of the points above.

Software and code

Policy information about availability of computer code

Data collection	Images from the intravital experiment were collected and processed using Slidebook v6.0 (Intelligent Imaging Innovations). Images from in vivo 3D scanning experiment were collected and processed using ScanImage v5.6 (Scientifica). The colocalization was analyzed using MATLAB2023 (MathWorks). Otherwise, no special softwares were used for data collection.
Data analysis	All data were analyzed using GraphPad Prism 8.0.

For manuscripts utilizing custom algorithms or software that are central to the research but not yet described in published literature, software must be made available to editors and reviewers. We strongly encourage code deposition in a community repository (e.g. GitHub). See the Nature Portfolio guidelines for submitting code & software for further information.

Data

Policy information about availability of data

All manuscripts must include a data availability statement. This statement should provide the following information, where applicable:

- Accession codes, unique identifiers, or web links for publicly available datasets
- A description of any restrictions on data availability
- For clinical datasets or third party data, please ensure that the statement adheres to our policy

The authors declare that the data supporting the findings of this study are available within the paper and its supplementary information files. The Mass Spec data generated in this study have been deposited to the ProteomeXchange Consortium via the PRIDE partner repository with the dataset identifier PXD050718 [https://www.ebi.ac.uk/pride/]. The relevant raw data from each figure are provided in the Source Data file.

Research involving human participants, their data, or biological material

Policy information about studies with human participants or human data. See also policy information about sex, gender (identity/presentation), and sexual orientation and race, ethnicity and racism.

Reporting on sex and gender	This study involves the utilization of pooled normal plasma isolated from human blood, and primary human umbilical vein endothelial cells (HUVECs) prepared from umbilical cords. The study did not collect or report the information on sex and gender of the donors.
Reporting on race, ethnicity, or other socially relevant groupings	The study did not collect or report the information on race, ethnicity and other socially relevant groupings.
Population characteristics	The donors recruited from the campus of Tongji Medical College and affiliated Tongji Hospital were healthy young individuals, with age between 20-30.
Recruitment	To prepare plasma from venous blood, healthy adult volunteers, including both males and females, were recruited through email lists or flyers on the campus of Tongji Medical College. To prepare primary umbilical vein endothelial cells (HUVECs) from umbilical cords after delivery, healthy adult volunteers were recruited in the Ob-Gyn Department at the affiliated Tongji Hospital. Informed consents were obtained from all the donors. The procedures were described in our previous publications (Br J Pharmacol 2023, 180:287; Pharmacol Res 2021, 167:105540). The donors were recruited based on their self-reported healthy status and in-hospital medical evaluation. Any individuals with severe thrombotic or bleeding symptoms were excluded and thus self-selection bias, if any, is unlikely to affect the results.
Ethics oversight	The procedures were approved by the Ethics Review Board of Tongji Medical College, Huazhong University of Science and Technology.

Note that full information on the approval of the study protocol must also be provided in the manuscript.

Field-specific reporting

Please select the one below that is the best fit for your research. If you are not sure, read the appropriate sections before making your selection.

☒ Life sciences ☐ Behavioural & social sciences ☐ Ecological, evolutionary & environmental sciences

For a reference copy of the document with all sections, see nature.com/documents/nr-reporting-summary-flat.pdf

Life sciences study design

All studies must disclose on these points even when the disclosure is negative.

Sample size	The sample size was determined based on our previous similar studies utilizing similar approaches (Nat Commun 2017, 8:14151; Br J Pharmacol 2023, 180:287; Pharmacol Res 2021, 167:105540; Arterioscler Thromb Vasc Biol 2024, 44:e82; Blood 2015,125:710). No sample-size calculation was performed.
Data exclusions	No data were excluded from analysis in this study.
Replication	Independent attempts at replication were performed to confirm the reproducibility of this study. All attempts at replication were successful. The representative results in the figures were from at least 3 independent experiments with similar results.
Randomization	For the experiments involving the utilization of animals, the samples were randomly allocated into experimental groups. For other experiments, the samples were also randomly allocated into different groups where possible using a 'random grouping table'.
Blinding	The investigators were blinded to group allocation during data collection and analysis where possible.

Reporting for specific materials, systems and methods

We require information from authors about some types of materials, experimental systems and methods used in many studies. Here, indicate whether each material, system or method listed is relevant to your study. If you are not sure if a list item applies to your research, read the appropriate section before selecting a response.

Materials & experimental systems

n/a	Involved in the study
☐	☒ Antibodies
☐	☒ Eukaryotic cell lines
☒	☐ Palaeontology and archaeology
☐	☒ Animals and other organisms
☒	☐ Clinical data
☒	☐ Dual use research of concern
☒	☐ Plants

Methods

n/a	Involved in the study
☒	☐ ChIP-seq
☒	☐ Flow cytometry
☒	☐ MRI-based neuroimaging

Antibodies

Antibodies used	The following antibodies were used: Anti-FLAG tag (Cell Signaling Technology Cat# 8146), Rabbit anti-human HRG (GeneTex Cat# GTX131514), Alexa-488-conjugated goat anti-mouse IgG (Thermo Fisher Scientific Cat# A-10680), Alexa-647-conjugated goat anti-rabbit IgG (Thermo Fisher Scientific Cat# A78957), HRP-conjugated mouse-anti human HRG (Angio-Proteomie Cat# hAP-0331), Anti-HRG rabbit polyclonal antibody (BBI Life Sciences Cat# D161754), Anti-Antithrombin (Affinity Biosciences Cat# DF6470), CoralLite488-congugated goat anti-mouse IgG (Proteintech Cat# SA00013-1), CoralLite488-congugated goat anti-rabbit IgG (Proteintech Cat# SA00013-2), Rabbit anti-human antithrombin (Sangon Biotech, Cat# D120215), HRP-conjugated goat anti-rabbit IgG (Proteintech Cat# SA00001-2), Dylight-649-conjugated anti-CD42c antibody (Emfret Cat# X649), Alexa-488-conjugated 59D8 antibody (gift from Dr. Bruce Furie of Harvard Medical School and prepared in house), Rat anti-mouse HRG antibody (Angio-Proteomie Cat# mAP-0091), Anti-CD31 (BioLegend Cat# 102416), Mouse antibody against HRG (Santa Cruz Biotech Cat# SC-398239), Goat anti-mouse HRG (R & D Systems Cat# AF1905), Transferrin Rabbit polyclonal Ab (Abclonal Cat# A1448), Factor XII (F12) antibody (Cloud-Clone Cat# PAA677Mu01), HRP-conjugated rabbit anti-goat (Proteintech Cat# SA00001-4).
Validation	The validation of each primary antibodies, as well as their RRID profiles if available, were provided in below. (1) Anti-FLAG tag (RRID:AB_10950495), used for western blot in this study, with validation available on its official website (https://www.cellsignal.cn/products/primary-antibodies/dykddddk-tag-9a3-mouse-mab-binds-to-same-epitope-as-sigma-aldrich-anti-flag-m2-antibody/8146). (2) Rabbit anti-human HRG (RRID:AB_2886495), used for western blot detection of human HRG in this study, with validation available on its official website (https://www.genetex.cn/Product/Detail/HRG-antibody/GTX131514). (3) mouse-anti human HRG, used for ELISA detection of human HRG in this study. The application in western blot and IHC were noted on its official website (https://www.angioproteomie.com/commerce/ccp2909-mouse-monoclonal-anti-human-hprg-hap-0331.htm). ELISA was validated in this study. (4) Anti-HRG rabbit polyclonal antibody, used for immunofluorescent detection of human HRG in this study. Its application in IHC detection of human samples was validated on its official website (https://store.sangon.com/productDetail?productInfo.code=D161754) (5) Anti-Antithrombin (RRID:AB_2838432), used for in vitro immunofluorescent detection of human and mouse antithrombin and in vivo immunofluorescent detection of mouse antithrombinin in this study. Its application in IHC and western blot detection of human, mouse and rat samples was noted on its official website (https://www.affbiotech.cn/goods-5272-DF6470-SERPINC1_Antibody.html) (6) Rabbit anti-human antithrombin, used for ELISA detection of human antithrombin and IHC detection of mouse antithrombin. Its application in ELISA and IHC detection of human sample was noted on its official website (https://store.sangon.com/productDetail?productInfo.code=D120215). (7) Dylight-649-conjugated anti-CD42c antibody (RRID:AB_2861336), used for in vivo labeling of platelets in this study. (https://www.emfret.com/fileadmin/user_upload/Datasheets/X649.pdf) Its validation available in our previous publications (Nat Commun 2017, 8:14151; Pharmacol Res 2021, 167:105540; Br J Pharmacol 2023, 180:287; J Am Chem Soc 2023, 145:3196). (8) Alexa-488-conjugated 59D8 antibody, prepared in house, used for in vivo labeling of fibrin in this study. Its validation is available in our previous publications (Nat Commun 2017, 8:14151; Pharmacol Res 2021, 167:105540; Br J Pharmacol 2023, 180:287; J Am Chem Soc 2023, 145:3196). (9) Rat anti-mouse HRG antibody, used for in vivo immunofluorescent detection of mouse HRG in this study. Its application in western blot detection of mouse HRG was noted on its official website (https://www.angioproteomie.com/commerce/ccp2243-rat-monoclonal-anti-mouse-hprg-map-0091.htm) (10) Alexa Fluor 647 anti-mouse CD31 Antibody (RRID:AB_493410), used for in vivo labeling of endothelial cells in this study. (https://www.biolegend.com/en-us/products/alexa-fluor-647-anti-mouse-cd31-antibody-3092) Its in vivo application was validated in a previous study (Nat Commun 2020, 11:1104). (11) Mouse antibody against HRG, used for immunofluorescent and IHC detection of mouse HRG in this study. Its application in western blot, immunofluorescence, IP and ELISA for both human and mouse samples was noted on the official website (https://www.scbt.com/p/hprg-antibody-g-10?requestFrom=search). (12) Goat anti-mouse HRG (RRID:AB_2121345), used for ELISA and western blot detection of mouse HRG in this study. Its application in western blot detection of mouse samples was noted on its official website (https://www.rndsystems.com/cn/products/mouse-hprg-antibody_af1905). The validation is available in a previous study (Biomaterials 2009, 30:3926). (13) Transferrin Rabbit Ab (RRID:AB_2761352), used for western blot detection of mouse transferrin in this study, with validation available on its official website (https://abclonal.com.cn/catalog/A1448). (14) Factor XII (F12) antibody, used for western blot detection of mouse FXII in this study, with validation available on its official website (https://www.uscnk.cn/uscn/Antibody-to-Coagulation-Factor-XII-F12-11883.htm).

Eukaryotic cell lines

Policy information about cell lines and Sex and Gender in Research

Cell line source(s)	The mouse brain microvascular endothelial cell line (bEnd.3) were purchased from AnweiSci (Shanghai), catalog#Bend3. Primary human umbilical vein endothelial cells (HUVECs) were prepared from umbilical cords collected after delivery at the affiliated Tongji Hospital. The primary HUVECs were pooled from different donors including both males and females. No sex information were collected.
Authentication	The authentication information about bEnd.3 cells (by STR profiling) were provided to the authors by the manufacturer. The authentication of primary HUVECs were available in our previous studies (Br J Pharmacol 2023, 180:287; Pharmacol Res 2021, 167:105540).
Mycoplasma contamination	All cell lines were tested negative for mycoplasma contamination.
Commonly misidentified lines (See ICLAC register)	None

Animals and other research organisms

Policy information about studies involving animals; ARRIVE guidelines recommended for reporting animal research, and Sex and Gender in Research

Laboratory animals	This study involves the FXII-deficient mice (F12-/-) and HRG-deficient mice (Hrg-/-). FXII and HRG double-knockout mice (DKO) were generated by cross breeding F12-/- with Hrg-/- mice. All mice were on a C57BL/6 background. Adult mice (8-16 weeks old) were used for experiments. The animals were housed in a standard facility with ambient temperature at 20-23 °C and humidity at 30-60% with 12 h light/dark cycles.
Wild animals	This study did not involve wild animals.
Reporting on sex	The findings apply to both sexes. Sex-based analysis was not performed since sex was not considered as an influencing factor in the study design.
Field-collected samples	This study did not involve samples collected from the field.
Ethics oversight	All animal care and procedure were approved by the Institutional Animal Care and Use Committee of Huazhong University of Science and Technology (HUST), and in accordance with the Guide for the Care and Use of Laboratory Animals promulgated by the National Institutes of Health.

Note that full information on the approval of the study protocol must also be provided in the manuscript.

Plants

Seed stocks	This study did not involve plants.
Novel plant genotypes	This study did not involve plants.
Authentication	This study did not involve plants.
